# Optimization of a general‐purpose, actively scanned proton beamline for ocular treatments: Geant4 simulations

**DOI:** 10.1120/jacmp.v16i2.5227

**Published:** 2015-03-08

**Authors:** Pierluigi Piersimoni, Adele Rimoldi, Cristina Riccardi, Michele Pirola, Silvia Molinelli, Mario Ciocca

**Affiliations:** ^1^ Department of Physics University of Pavia Pavia Italy; ^2^ INFN, Section of Pavia Pavia Italy; ^3^ Department of Radiation Research Loma Linda University Loma Linda CA USA; ^4^ Medical Physics Unit, CNAO Foundation Pavia Italy

**Keywords:** proton interaction in matter, proton therapy, Geant4 simulation, CNAO, uveal melanoma

## Abstract

The Italian National Center for Hadrontherapy (CNAO, Centro Nazionale di Adroterapia Oncologica), a synchrotron‐based hospital facility, started the treatment of patients within selected clinical trials in late 2011 and 2012 with actively scanned proton and carbon ion beams, respectively. The activation of a new clinical protocol for the irradiation of uveal melanoma using the existing general‐purpose proton beamline is foreseen for late 2014. Beam characteristics and patient treatment setup need to be tuned to meet the specific requirements for such a type of treatment technique. The aim of this study is to optimize the CNAO transport beamline by adding passive components and minimizing air gap to achieve the optimal conditions for ocular tumor irradiation. The CNAO setup with the active and passive components along the transport beamline, as well as a human eye‐modeled detector also including a realistic target volume, were simulated using the Monte Carlo Geant4 toolkit. The strong reduction of the air gap between the nozzle and patient skin, as well as the insertion of a range shifter plus a patient‐specific brass collimator at a short distance from the eye, were found to be effective tools to be implemented. In perspective, this simulation toolkit could also be used as a benchmark for future developments and testing purposes on commercial treatment planning systems.

PACS numbers: 21.30Fe, 24.10.Lx, 29.20.dk, 29.27.Eg, 29.85.Fj

## I. INTRODUCTION

The ocular melanoma is currently the most common early intraocular tumor in adulthood, with a relatively low incidence rate per year. Uveal melanoma is a malignant tumor which tends to grow both inside the bulb, invading and disrupting the intraocular tissues, and outside it, infiltrating the sclera and orbital tissues. With regard to the radiation treatment options for this disease, proton therapy and brachytherapy with plaques are both considered today valid and conservative alternatives to radical eye surgery enucleation, and offer the patient the possibility to maintain a good quality of life. Furthermore, these types of treatments allow, in most cases, the retention of residual visual capacity of the eye involved by the neoplasia. In particular, proton radiotherapy for ocular melanoma results in an overall very satisfying local control of 97% at 5 yrs, 96% at 10 yrs, 94% at 15 yrs, and overall tumor specific survival rates of 91% at 5 yrs, 83% at 10 yrs, and 79% at 15 yrs. Differentiated outcome analysis shows that age, tumor size (diameter and thickness), localization, and relation to other structures (optic disc, ciliary body, iris) have the strongest influence on local failure, enucleation rate, and survival.^1^, ^2^

Good results have been obtained in Italy at the INFN (Istituto Nazionale di Fisica Nucleare) Laboratori Nazionali del Sud, within the “CATANA proton therapy” project, where more than 200 patients have been treated since 2002.^(3)^ Several other experiences in eye proton radiotherapy have already been implemented worldwide, mostly using a dedicated beamline, rather than a large‐field and general‐purpose one.^(4)^ For example, in Europe, at Paul Scherrer Institute (Switzerland), since 1984 more than 4,700 patients were treated within the OPTIS Program (Proton therapy for tumors of the eye).^(5)^ The Orsay Proton Therapy Center (CPO), France, was created in January 1991 and started the first ophthalmological treatments in April 1991. Between 1991 and 2010 more than 5,000 patients were treated, in 80% of cases for eye melanoma.^(6)^ In the same years at the Medicyc Cyclotron 65 MeV proton beam facility in Nice, France, almost 4,000 patients were treated for uveal melanomas.^(7)^

The Italian Centro Nazionale di Adroterapia Oncologica (CNAO) was opened in Pavia in 2010 and, since then, more than 200 patients have been treated with actively scanned proton and carbon ion pencil beams. The core of this high‐technology advanced facility is a synchrotron designed for medical purpose, able to accelerate both protons and carbon ions to very high energies. In late 2011, a clinical experimentation phase, successfully completed at the end of 2013, started at CNAO with proton and carbon ion treatments for deep‐seated tumors, like as chordoma and chondrosarcoma of the skull base and spine or sarcoma of head and neck.^(8)^ These kinds of tumors have different characteristics with respect to the very small sized and quasi‐superficial uveal melanomas in the eye. However, an ongoing project, focused on the customization of one of the three available fixed transport beamlines, is planned in order to make the CNAO accelerated beam also suitable for ocular tumor treatments. The optimization of the CNAO transport beamline for such type of treatment will be discussed in this work, by modeling each component using a Monte Carlo simulation.

Several Monte Carlo studies have been published about proton radiotherapy for uveal melanoma, using different codes (e.g., MCNPX code).^9^, ^10^, ^11^, ^12^ In this work, the Geant4^13^, ^14^, ^15^, ^16^ open‐source toolkit, entirely written in the Object Oriented language C++, has been used. The main advantage of using Geant4 is represented by its versatility, as well as its great power in simulating very complex geometries such as a human eye. Moreover, Geant4 satisfies one of the most critical requirements in every Monte Carlo simulation, that is to provide a highly reliable description of all the physical processes at the energies relevant to the application under study. The Geant4. 9.6 release has been used for the application described in this work.

The use of radiation therapy for intraocular tumors started since the early 20th century. Several possible modalities are available (e.g., conventional radiotherapy, brachytherapy, proton therapy) and controversies continue to exist regarding the most appropriate radiotherapy modality to treat uveal melanoma. As demonstrated by Weber et al.^(17)^ the use of photon beam stereotactic techniques, compared with protons, can result in similar levels of dose conformation. However, thanks to the physical properties of hadrons penetrating matter, proton or heavier ion treatments can improve the visual prognosis, because the energy is delivered to the target, with very little exposure of surrounding healthy tissues.^(18)^

In the clinical practice of eye proton therapy, the currently worldwide accepted technique for dose delivery is represented by the passive scattering modality,^19^, ^20^ while the treatment plans are usually calculated using the EYEPLAN software, developed in 1983 by Goitein and Miller^(21)^ and subsequently adapted by Sheen (Douglas Cyclotron, Clatterige Center for Oncology, UK). Low‐energy cyclotrons (62–72 MeV) or high‐energy (up to approximately 250 MeV) cyclotrons or synchrotrons can be used.^(4)^ A planning study with carbon ions has been also reported for eye treatment at NIRS, Japan, using an in‐house treatment planning software: NIRS‐EYEPLAN. This program has functionalities of both 3D CT‐based planning and eye modeling. The treatment technique consists of orthogonal two‐port arrangement coupled with the use of a range compensator that shapes the range of the broad beams to the distal periphery of the tumor volume.^(22)^

In the present configuration at CNAO, however, the dose delivery system adopted, and only available to treat patients, is represented by the full active scanning modality (the so‐called pencil beam scanning),^8^, ^23^ and a commercial general‐purpose, image‐based treatment planning system is used. The aim of this work is to optimize the existing, nondedicated transport beamline in the perspective of extending its use also to ocular treatments.

## II. MATERIALS AND METHODS

### A. MC Simulation and geometry setup

The CNAO synchrotron is able to produce proton beams with a FWHM ranging from a minimum of 3.5±0.4 mm to a maximum of 5.4±0.5 mm at the vacuum exit window and from 7.0±0.7 mm to 22.3±0.2 mm at the isocenter, inversely depending on the beam energy. The available energy range for proton beams is 63–250 MeV, with an uncertainty of 0.05%. Carbon ion beams (120–400 MeV/u energy range) can also be produced, but they are out of the scope of this work.

**Figure 1:**
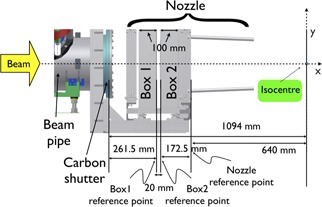
Schematic side view of the final part of the CNAO beam transport line. From the left‐hand side, the following components are respectively shown: the beam pipe, the carbon fiber exit window, and the nozzle structure inside of which the two beam monitoring chambers (Box 1 and Box 2) are located.

The final part of the standard transport beamline set at CNAO^(8)^ is shown in Fig. 1. Projectile charged particles are accelerated in the CNAO synchrotron ring, travel in a long extraction vacuum beam pipe, crossing the magnetic field generated by two orthogonal defecting magnets (not described in this paper). The vacuum beam pipe is sealed by a carbon fiber exit window, after which the accelerated beam reaches the treatment room and travels for several centimeters in air. In the treatment room the beam crosses a fixed structure called nozzle, inside of which two beam‐monitoring chambers (Box 1 and Box 2) are embedded. These chambers are used to measure, in real time, the beam fluence and position. The standard isocenter is located at a distance of 64 cm from the downstream edge of the nozzle.

The full CNAO transport beamline with all its elements was simulated using the Geant4 toolkit. The geometry for the beam delivery line was built using the standard Geant4 approach. Readout geometry was used for scoring purposes, as well as parallel geometries (scoring volumes), for a further check of the obtained results.

The simulation of the transport beamline consisted of all the elements shown in Fig. 1. All the simulated elements were positioned in an air mother volume (not visible in Fig. 1). The beam was fired inside a vacuum beam pipe, 6 m long (the last section is visible in the left hand side of Fig. 1). All the transport beamline components were built using standard materials.

The most complex elements along the transport beamline are the monitoring chambers, Box 1 and Box 2. Their internal structure^(24)^ was studied in order to reproduce them as accurately as possible in the Geant4 implementation. They are gaseous $N_{2}$‐filled parallel plate ionization chambers. Inside of Box 1, an assembly of two different devices is present: an integral chamber to measure beam fluence and two strip chambers to measure the beam position in the vertical and horizontal directions. Box 2 includes an integral chamber (similar to one inside Box 1) to measure the beam fluence and a pixel chamber which measures the beam position on the transverse plane.

**Figure 2:**
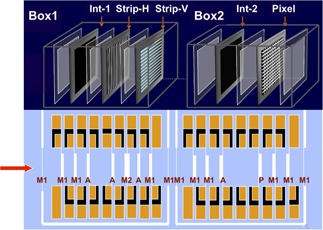
Inside view of the two monitoring chambers (Box 1 and Box 2). The red arrow indicates the particle beam direction. (top) The internal structure of the boxes with integral, strip and pixel detectors. (bottom) The thin layers composing the ionization chambers and their arrangement inside the Al frame.

The internal structure of the boxes (Fig. 2) consists of segmented anodes mounted on A1 frames to guarantee stability and rigidity to the system. Anodes of each monitoring chamber are made of thin layers positioned at the correct depth inside the ionization chambers Box 1 and Box 2. They are of four different types:

i) M1 made of a 12 μm thick layer of Mylar (DuPont Teijin Films, Chester, VA) and a 1 μm thick layer of Al;
ii) M2 made of a 25 μm thick layer of Mylar and a 2 μm thick layer of Al;
iii) A made of a 25 μm thick layer of Kapton (DuPont Teijin Films) and a 10 μm thick layer of Al;
iv) P made of a 50 μm thick layer of Kapton and a 20 μm thick layer of Cu.

M1 is used as shutter and integral cathode in both chambers, M2 is used as cathode strip in Box 1, A is used as integral anode in both boxes and as anode strip in Box 1, P is used as pixel anode in Box 2. The compositions and the positioning of these layers have been implemented in deep detail in the simulation, avoiding any possible approximation.

The proton beam within the simulation was fired along the X direction, and it was generated with an energy spread of 0.05% and a spatial spread symmetric in the Y and Z directions varying from 3.77 mm to 5.42 mm, inversely depending on the nominal particles energy. These parameters were set in agreement with the CNAO synchrotron features.

Four different options for the incoming beam are provided: a) point‐like; b) bidimensional grid with horizontal and vertical beam defection (Y and Z axes); c) energy scan in the beam direction (x‐axis) for in‐depth scans; and d) the two previous modalities coupled, in order to reproduce the active scanning modality adopted at CNAO.

The Geant4 application has been built following the advanced/hadrontherapy example of the Geant4 distribution.^(25)^ Following authors' suggestions, the following physics packages were activated: a) G4EmStandardPhysics_option3 for the electromagnetic model; b) G4HadronElasticPhysics for the hadronic elastic model; c) G4HadronInelasticQBBC for the hadronic inelastic model; d) G4RadioactiveDecayPhysics for the radioactive decay.

For the electromagnetic model different processes were activated for different particles. The list below describes the C++ classes used to simulate the physics processes for each particle:

– gamma: G4ComptonScattering, G4PhotoElectricEffect, G4ComptonScattering, G4RayleighScattering
– e‐: G4eMultipleScattering, G4eIonisation, G4Bremsstrahlung according to the G4UrbanMscMode195
– generic ion: G4ionIonisation, G4hMultipleScattering, G4ionIonisation, G4NuclearStopping according to G4IonParametrisedLossModel
– p: G4hMultipleScattering, G4hIonisation, G4hBremsstrahlung, G4hPairProduction, G4NuclearStopping

For the hadronic elastic physics, the Chiral Invariant Phase Space (CHIPS) model for sampling scattering for p and n was adopted (G4CHIPSElastic class), while the Low and High Energy Parameterized (LHEP) sampling model for the other particles was used (G4HadronElasticProcess class). The G4ChipsProtonElasticXS, G4NeutronElasticXS, and G4BGGPionElasticXS classes were used to calculate the cross‐sectional formulas for p, n, and $\pi^{\pm }$, respectively, while the LHEP cross‐sectional formula was used for other particles (G4CrossSectionElastic class).

For the hadronic inelastic physics, the following classes were activated for p and n:

– p: G4PreCompoundModel, G4CascadeInterface, G4BinaryCascade using the Barashenkov‐Glauber cross section formula (G4BGGNucleonInelasticXS class)
– n: G4CascadeInterface, G4BinaryCascade, G4NeutronRadCapture using the neutron cross section formula (G4NeutronInelasticXS class)

Further details about the physics lists can be found in the Geant4 literature.^(26)^

The Bragg peak position for the minimum energy deliverable by the CNAO synchrotron (63 MeV) is 3 cm in water, potentially falling beyond the anatomical depth of the eye. A degradation of the proton energy using a PMMA range shifter is, therefore, required. The correct range shifter thickness must be evaluated case by case in order to obtain the desired energy degradation. A simple $25\times 25\times 25\;{\text{mm}}^{3}$ water box detector was used to study the dependence of the proton range on the range shifter thickness.

An unwanted effect of the introduction of the range shifter is represented by the broadening of the transverse beam profile because of the Coulomb scattering of proton crossing the PMMA. To reduce this effect, it was decided to shift the isocenter for the ocular tests upstream with respect to the standard isocenter adopted at CNAO. Together with the CNAO medical physics and bioengineering staff, it was decided to fix the new isocenter 11 cm downstream from the end of the nozzle. In this way, the beam is prevented to cross several centimeters of air, while the patient can be comfortably seated on the treatment chair and have enough air gap to gaze at a fixed point for precision alignment purposes.

### B. Eye simulation

**Figure 3:**
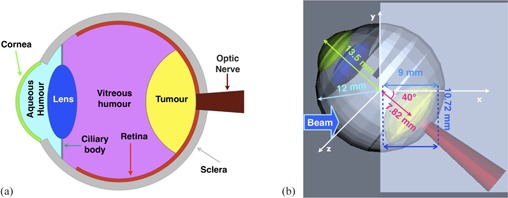
(a) Schematic side view of the Geant4 simulated eye. b) Side view of the eye‐detector implemented in the Geant4 simulation when rotated up by 40° for irradiation purposes. A dome‐shaped volume inside the eye (yellow) mimics the tumor extending in depth from the isocenter to 9 mm after it, while the red volume on the right‐hand side is the simulated optical nerve. The eye protrudes from the brain (a water box of 48 mm side), here in light blue. Green and blue structures on the left represent cornea and lens, respectively. Other crucial subcomponents (retina, vitreous humor, aqueous humor) are set invisible in order to make the picture clearer.

In this simulation, a detailed description of the human eye with its internal components was used to build a detector. A realistic tumor was also included. Figure 3(a) shows a simplified human eye structure used to reproduce each eye component in a geometrical shape which can be implemented by the Geant4 code. Figure 3(b) shows a three‐dimensional visualization of the eye detector in the frame of reference of the simulation. The main dimensions used in the eye modeling are reported. The eye was put in a water box mimicking a human brain with 48 mm side, protruding from it in an aspect‐ratio similar to a human head.

For the eye component materials implementation, a two‐step approach was used. Firstly, a list of custom materials was created to reproduce all the chemicals present in a human eye (Table 1). Secondly, these elementary materials were used in association to form the final material for each eye component (Table 2). Information about eye anatomy and eye component composition was obtained from ophthalmological literature.^(27)^ Moreover, all the eye dimensions have been implemented as parameterized as a function of the outer sclera radius. In this way it is simple to rescale the eye according to the size of different patients' eyes.

**Table 1:** Materials and relative chemical compositions used to simulate the eye detector. Collagen is a mixture of two simpler materials: proline and idrossiproline. For proteins, a chemical composition corresponding to the average of the chemical composition of the 20 amino acids is used. To each material a default density of 1 g/cm^3^ is assigned

*New Material*	*Composition*
Proline	H_9_C_5_O_2_N_1_
Idrossiproline	H_9_C_5_O_3_N_1_
Collagen	Proline (86%) + Idrossiproline (14%)
Lipids	H_48_C_24_O_6_P_1_N_2_
Lactate	H_5_C_3_O_2_
Sugar	H_2_C_1_O_1_
N‐AcetilAspartate (NAA)	H_9_C_6_O_5_N_1_
Choline	H_14_C_5_O_1_N_1_
Creatine	H_9_C_4_O_2_N_3_
Proteins	H(50%) + C(28%) + O(13%) + N(8%) + S(1%)

**Table 2:** Eye components, corresponding materials with percentage proportion (fraction of mass) and densities used in the simulation of the eye detector

*Eye Component*	*Material*	*Density (g/cm^3^)*
Aqueous Humor	H_2_O (98.5%) + NaCl (1.5%)	1.0080
Vitreous Humor	H_2_O (98.5%) + Protein (1.5%)	1.0050
Sclera, Cornea, Ciliary Body	Collagen (50%) + Protein (25%) + Sugar (25%)	1.0710
Crystalline Lens	H_2_O (60%) + Protein (40%)	1.0670
Retina	H_2_O(80%) + NAA (10%) + Choline (5%) + Creatine (5%)	1.0174
Tumor	H_2_O (80%) + NAA (3%) + Choline (12%) + Creatine (3%) + Lipids (1%) + Lactate (1%)	1.0174

Each eye element was made sensitive in the Geant4 simulation and separate hit collections, one for each of them, were created in order to evaluate the dose deposition on the different tissues.

To avoid undesired dose to the healthy eye structures (in particular the cornea and the lens), in the simulation is possible to misalign tumor as much as possible from these eye components, by rotating the eye detector. In Fig. 3(b) the detector is shown rotated by an angle of 40° in the vertical direction. Due to this rotation the simulated tumor is shifted down from the beam direction (x‐axis) by a certain amount, consequently the eye detector during the irradiation must be translated by the same amount in order to still have the tumor center along the beam central axis. In a real treatment, to have the eye rotated in the position suggested, the patient would be supposed to gaze at the same fixed point for the total irradiation time needed for a daily treatment fraction.

**Figure 4:**
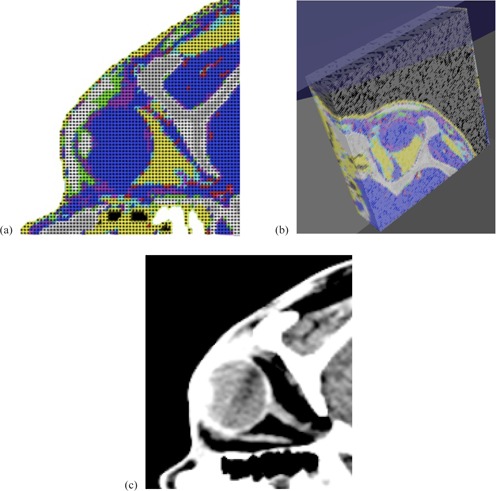
Top view (a) and 3D view (b) of the implemented DICOM detector and the reference CT image (c) as displayed by a commercial DICOM image viewer.

As a further development of the studies reported in this paper, an example was added in order to show the possibility to import medical images in the standard DICOM format^(28)^ and use their information as possible target volumes. In this example, ten contiguous slices from a CT scan of a human head were used. The original slices had a granularity of $512\times 512\;\text{pixels},0.97\times 0.97\;{\text{mm}}^{2}$ per pixel, each slice corresponding to a thickness of 2 mm. Only an inner portion of 86×86 pixels from each original slice, containing the right eye, was selected. With this information directly read from DICOM files, a new simulated detector was built (Fig. 4).

### C. Validation of the simulation against experimental data

The simulation was validated against experimental results obtained at CNAO in order to check whether the simulated setup well reproduced the standard experimental setup. Following the simulation of the standard CNAO transport beamline, the transverse FWHM of the beam was measured at five different points along the transport beamline, for four different beam nominal energies. For this purpose, a thin sensitive air layer detector (1 mm thick), simulating radiochromic films for radiation dosimetry, was used. Moreover, the depth‐dose distribution and proton range in a water phantom by simulated beams with four different nominal beam energies were calculated.

### D. Uniform irradiation of the target volume

According to a realistic clinical scenario, a uniformly irradiated cubic volume was simulated, using a 3 mm scanning step in the transverse plane, over a square field size of $40\times 40\;{\text{mm}}^{2}$ centered on the tumor, and a spread‐out Bragg peak (SOBP) width approximately equal to a typical uveal melanoma longitudinal dimension. Energy layers were optimized to obtain a uniform dose distribution within the tumor volume.

To achieve a sharper lateral dose gradient, a brass collimator, 10 mm thick, was inserted in the transport beamline. The collimator aperture was set as big as the tumor transverse cross section plus 3 mm margin. The choice of brass came from its widespread use in proton radiotherapy with passive scattering technique. The simulated material for the brass collimator is an alloy of 61.5% of copper, 35.2% of zinc, and 3.3% of lead.

After the optimization of the energy deposition in the simple water box detector, almost the same beam setup was used to irradiate the eye detector and the DICOM detector. Because of the different geometry of the two detectors it was found that for the DICOM detector a thicker range shifter was necessary.

## III. RESULTS

### A. Comparison between simulated data and CNAO experimental results

In Table 3, a comparison between simulated data and CNAO‐measured data with relative uncertainties is reported. The experimental data were measured with radiochromic films and uncertainties were evaluated to be about 10% of each obtained value. Simulated data were produced generating $2.5\times 10^{5}$ events in five statistical independent runs. Uncertainties were evaluated as square root of unbiased sample variances.

**Table 3:** FWHM in the transverse plane measured at CNAO and with the Geant4 simulation. For each energy the detector was positioned at five different positions along the transport beamline: at the exit window (−1093.6 mm upstream from the isocenter), after the monitoring chamber Box 2 (−654.6 mm), at an intermediate point (−350 mm), at the isocenter (0 mm), and at a position beyond the isocenter (500 mm)

*Position (mm)*	*Nominal Energy (MeV)*				
	*81.56*	*100.51*	*119.05*	*148.80*	
−1093.6	5.48±0.55	5.60±0.56	5.50±0.55	4.05±0.41	CNAO FWHM
−654.6	8.35±0.84	7.24±0.72	6.60±0.66	5.65±0.57	
−350.0	12.52±1.25	10.46±1.05	9.13±0.91	7.44±0.74	
0.0	16.39±1.64	13.63±1.36	11.25±1.13	9.80±0.98	
500.0	24.52±2.45	20.64±2.06	17.70±1.77	14.15±1.41
−1093.6	5.73±0.013	5.71±0.01	4.99±0.01	4.96±0.01	Geant4 FWHM
−654.6	7.94±0.03	6.91±0.01	6.67±0.01	5.84±0.01	
−350.6	11.62±0.03	9.08±0.02	9.55±0.02	7.53±0.02	
0.0	16.67±0.03	12.51±0.02	13.80±0.02	10.19±0.02	
500.0	24.13±0.04	17.86±0.31	20.28±0.03	14.66±0.02

**Figure 5:**
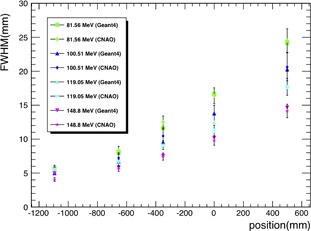
Comparison between measured and simulated beam FWHM vs. detector positions at four proton beam nominal energies (81.56 MeV, 100.51 MeV, 119.05 MeV, and 148.8 MeV). For the definition of the five detector positions, please refer to Table 3 caption. A parallel geometry superimposed to the detector was used for scoring purpose. The parallel geometry consists of a grid containing 150×150×150 voxels, each with dimension $0.25\times 0.25\times 0.25\;{\text{mm}}^{3}$.

In Fig. 5, a comparison of the simulated and measured FWHM plotted against the detector position is shown. Experimental data and simulation data overlap within the experimental uncertainties, therefore the agreement was found to be good. Uncertainties shown in Fig. 5 refer to experimental data points. Simulated data uncertainties are included in the point markers.

**Figure 6:**
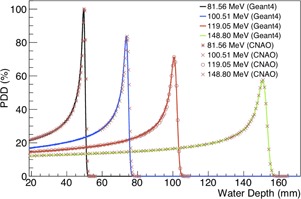
Percentage depth‐dose distributions in a water phantom obtained for proton beams with four different nominal energies. Curves are normalized at the dose deposited by the beam with lower energy (81.56 MeV).

Percentage depth‐dose distributions and extrapolated proton ranges in a water phantom measured at CNAO were compared with the same set of simulated data (Table 4). Experimental data were measured using a peak‐finder device (http://www.ptw.de/peakfinder.html). Also, in this case, the simulation was performed by generating $2.5\times 10^{5}$ events in five statistical independent runs. The spatial resolution of the measured data is 0.01 mm, while in the simulation a read out geometry with a pixel of 0.05 mm was used. Uncertainties reported in Table 4 for the simulated data account for both systematic and statistic errors. In Fig. 6, a comparison of the simulated and measured FWHM plotted against the detector position is shown. The discrepancy between simulation and experimental data is lower than 1%, hence the agreement, again, can be considered nice.

**Table 4:** Proton range (depth penetration in water) for beams with four different nominal energies measured at CNAO and by using the Geant4 simulation

*Beam Nominal Energy (MeV)*	*CNAO Proton Range in Water (mm)*	*Geant4 Proton Range in Water (mm)*
81.56	50.00±0.01	49.65±0.1
100.51	74.00±0.01	73.60±0.1
119.05	101.00±0.01	100.65±0.1
148.8	151.00±0.01	150.65±0.1

### B. Degradation of the CNAO proton beam energy

Since the CNAO synchrotron is optimized for high‐energy treatment, it is better performing at energies greater than 80 MeV. For this reason it was decided to use an energy of 100.51 MeV for the subsequent tests. A beam with such an energy must be degraded with a range shifter with an opportune thickness, to be used in eye protontherapy.

**Figure 7:**
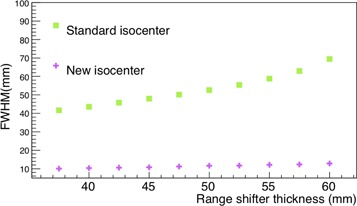
Beam FWHM in the transverse plane at the standard CNAO isocenter (green square markers) and at the optimized isocenter (magenta cross markers) for a proton beam with a nominal energy of 100.51 MeV vs. different values of range shifter thicknesses. Uncertainties are included in the point markers.

The dependence of the beam FWHM on the range shifter thickness for a beam with a nominal energy of 100.51 MeV was studied with a thin air layer detector positioned at the isocenter and the range shifter at a distance of 65 mm upstream the isocenter. The beam transverse FWHM was plotted versus the range shifter thickness data and it ranges from 40 mm to 70 mm (green square markers Fig. 7). The FWHM measured in these conditions, then, is at least four times larger than the beam FWHM without the range shifter (see Table 3), even for not very high thickness (40 mm), and it results to be bigger than a human eye‐section diameter. To reduce the FWHM broadening effect, the detector was positioned at the new isocenter, at 11 cm downstream the nozzle. The beam FWHM on the transverse plane was measured at the new isocenter, for different values of range shifter thickness, positioned at 65 mm upstream the new isocenter. The beam FWHMs measured in these conditions (magenta cross markers in Fig. 7) go from 10 to 13 mm. These values are consistent with the standard CNAO pencil beam, which has a FWHM in the transverse plane of about 13 mm for a nominal beam energy of 100.51 MeV (see Table 3). For both the series of data in Fig. 7, 10^5^ primary events were simulated.

### C. Uniform irradiation of the target volume

The target volume (i.e., the tumor in the eye detector) should be uniformly irradiated in the three dimensions. Transverse and in‐depth uniform irradiations were separately treated.

#### C.1 Uniform irradiation on a transverse plane

**Figure 8:**
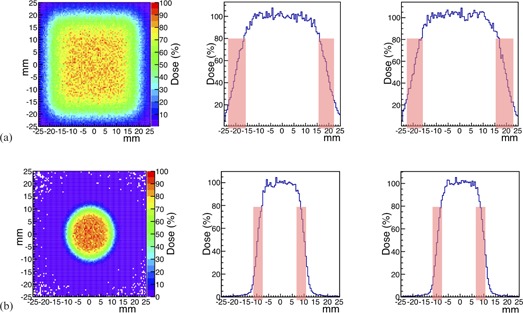
a) Transverse beam uniformity scan obtained with a monochromatic beam of 100.51 MeV degraded with a PMMA range shifter 43 mm thick. Left‐hand side: dose distribution on the YZ plane normalized on the maximum value. Center and right‐hand side: beam profile along Y and Z directions, respectively, normalized with respect to the central value. b) Transverse beam uniformity scan obtained with a monochromatic beam of 100.51 MeV degraded with a PMMA range shifter 43 mm thick and the addition in the beamline of a brass collimator 1 cm thick. Left‐hand side: dose distribution on the YZ plane normalized on the maximum value. Center and right‐hand side: beam profile along Y and Z directions, respectively, normalized with respect to the central value.

A uniform irradiation in the YZ plane was simulated to mimic the work done by the scanning magnets at the CNAO facility. The active scanning dose delivery system at CNAO works by changing the beam transverse direction in discrete steps of 3 mm. Uniformity in the transverse plane was obtained in the simulation with the same configuration. In Fig. 8(a) from left to right, respectively, a bidimensional profile corresponding to a $40\times 40\;{\text{mm}}^{2}$ scan with steps of 3 mm, the beam profile in the Y and in the Z directions are shown. The simulation was realized with $1.8\times 10^{6}$ simulated events, generated in 2 statistical independent runs, at an energy of 100.51 MeV and degraded with a PMMA range shifter 43 mm thick, positioned at 65 mm upstream the new isocenter. The dose distribution is normalized to its maximum value. In the central Y and Z profiles zone, between about −16 mm and 16 mm, where the dose is higher than the 80% of the central value, the dose values are distributed in a Gaussian shape with a uniformity of about 7% for both the Y and Z profile. The values of the lateral penumbra (that is the falloff of dose between 80% and 20%) are reported in Table 5. The values refer to the energy deposited in the central region (0.5 mm thick) of a thin water layer detector (1 mm thick) positioned at the optimized isocenter.

To reach uniformity in a surface area as big as the tumor lateral section, a brass collimator with a 10 mm aperture was added in the beamline, just after the range shifter, at 50 mm from the isocenter. In Fig. 8(b) from left to right, the dose distribution on the transverse YZ plane of $40\times 40\;{\text{mm}}^{2}$ beam scan with steps of 3 mm, the beam profile in the Y direction and the beam profile in the Z direction are shown. The dose distribution profile is normalized to its maximum value. In the central Y and Z profiles zone, between about −7 mm and 7 mm where the dose is higher than the 80% of the central value, the dose values are distributed in a Gaussian shape with a uniformity of about 4% for both the Y and Z profiles. The lengths of the lateral penumbra are reported in Table 5. These values refer to the energy deposited in the central region (0.5 mm thick) of a thin water layer detector (1 mm thick) positioned at the isocenter.

**Table 5:** Lateral penumbra for the Y and Z profile, obtained with a simulated irradiation on the transverse plane YZ

	*80‐20% Lateral Penumbra (mm)*	
	*Y*	*Z*
No Collimator	7.25±0.07	6.50±0.07
Collimator In	4.00±0.07	3.50±0.07

#### C.2 Spread‐out Bragg peak

**Figure 9:**
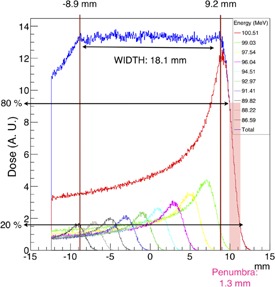
The SOBP (blue curve) obtained with the simulated beamline as a weighted sum of 10 energy distributions (colored curves), irradiating a water box $\left(25\times 25\times 25\;{\text{mm}}^{3}\right)$. A parallel geometry was superimposed on the water box in order to score the energy deposition on the target. The parallel geometry (slightly bigger than the target volume) consists of 600×30×30 voxels, each one of $0.05\times 1\times 1\;{\text{mm}}^{3}$ volume. The 0 corresponds to the isocenter, as well as the center of the water box.

An SOBP was created in order to have a uniform irradiation all over the beam propagation direction. An algorithm was adopted to calculate the correct weights ($w_{j}$) to be used for the SOBP. Each weight was calculated using the following formula:

(1) $$w_{j}=\frac{1}{H}-\sum_{k=1}^{j-1}\frac{h_{k}(b_{j})}{H}$$

where *H* represents the arbitrary maximum height to be reached, $h_{k}$ is the height of the beam with the kth energy that produces a Bragg peak in the bin $b_{j}$. The set of chosen energies is shown in Table 6. The range shifter, with the optimized thickness for a nominal beam energy of 100.51 MeV, was added in the beam path at a distance of 65 mm from the isocenter. The simulated SOBP was produced inside a simple water cube detector $\left(25\times 25\times 25\;{\text{mm}}^{3}\right)$ positioned at the new isocenter. The obtained SOBP (Fig. 9) inside of the water cube resulted to have a width of 18.10±0.02 mm, extending from −8.9 mm upstream the new isocenter to 9.2 mm downstream the new isocenter. The peak/entrance ratio resulted 80% and the distal falloff of dose from 80% to 20% was 1.3±0.02 mm. The produced SOBP is just an example to test the algorithm implemented to build it. For the irradiation of a target volume, the correct number of energies to be used must be evaluated, in order to achieve the desired SOBP width.

**Table 6:** List of the proton beam energies, the proton penetration depths, and the weights used to produce a 18.2 mm SOBP using the Gean4 simulation. The depth penetration in water was measured for a proton beam degraded with a 43 mm thick range shifter (RS). To irradiate the simulated tumor in the eye detector, the first six energies listed were used

*Nominal Energy (MeV)*	*Depth in Water with RS (mm)*	*Weight (%)*
100.51±0.05	35.50±0.25	90.00
99.03±0.05	33.50±0.25	32.10
97.54±0.05	31.50±0.25	25.60
96.04±0.05	29.50±0.25	21.00
94.51±0.05	27.50±0.25	18.24
92.97±0.05	25.50±0.25	15.66
91.41±0.05	23.50±0.25	14.00
89.82±0.04	21.50±0.25	13.00
88.22±0.04	19.50±0.25	11.00
86.59±0.04	17.50±0.25	10.00

### D. Summary of the transport beamline optimization

**Figure 10:**
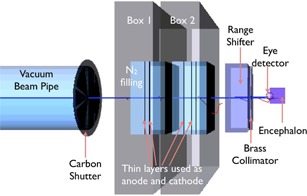
The CNAO transport beamline after the optimization for the eye treatment. In this optimization a PMMA layer (range shifter), for degrading the excess of delivered energy, and a brass collimator were added at short distance (65 mm and 50 mm, respectively) with respect to the detector (eye) positioned in the isocenter.

The PMMA range shifter has an optimized thickness of 43 mm. This value at the chosen energy of 100.51 MeV guarantees to reach both the tumor volume and the safety margin (3 mm).^(29)^ Because of the eye rotation, the tumor has an elliptical front section in the transverse plane. A brass collimator with a thickness of 10 mm and an ellipsoidal aperture of $20\times 22\;{\text{mm}}^{2}$ was added in the transport beamline just after the range shifter at 50 mm upstream from the isocenter, to better focus the dose deposition on the tumor. Mutual positioning of these passive elements is shown in Fig. 10.

### E. Irradiation of the eye detector

**Figure 11:**
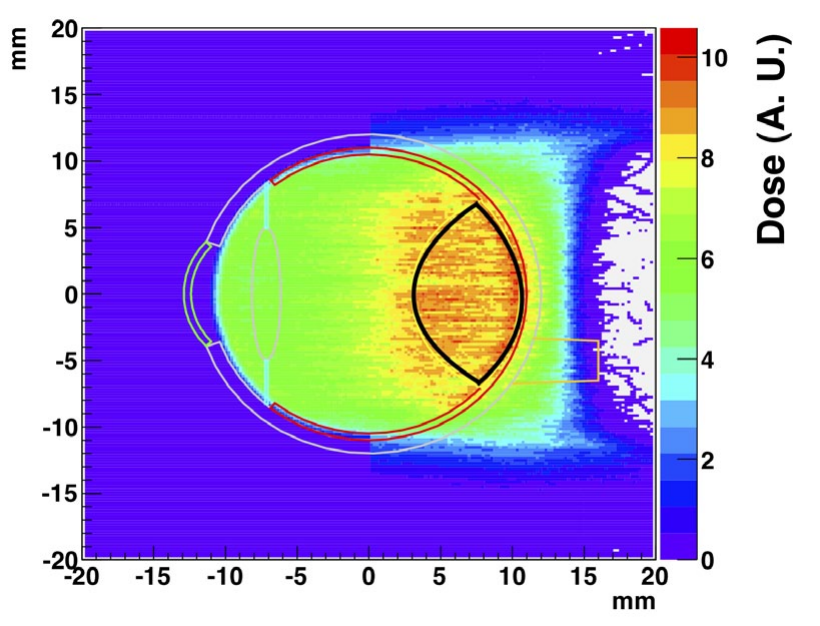
Top projection of the irradiated eye detector using a lateral scan of $40\times 40\;{\text{mm}}^{2}$ and six energies of SOBP (Table 6). The irradiation setup includes the PMMA range shifter (43 mm thick) and a brass collimator with an elliptical aperture of $22\times 20\;{\text{mm}}^{2}$ positioned, respectively, at 65 mm and 50 mm from the isocenter. Irradiation was performed simulating $3.6\times 10^{6}$ events.

With the setup just described, the treatment uniformity on the target (the tumor in the eye detector) was tested. In Fig. 11, the resulting dose distribution in an orthogonal plane (top projections) of the irradiated eye detector with $3.6\times 10^{6}$ events is shown. The beam is coming from the left side, as shown in Fig. 10. The tumor (black shape on the back portion of the eye) is uniformly irradiated all along its depth, thanks to the generated SOBP. To achieve a SOBP width of about 12 mm (necessary to achieve a uniform irradiation all along the simulated tumor depth), the first six energies listed on Table 6 were used. In the projection shown in Fig. 11, the dose deposited on the tumor is at least twice as the one deposited elsewhere.

**Figure 12:**
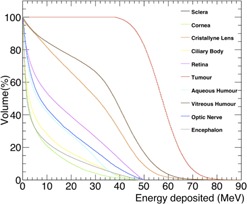
Comparative DVH for the simulated irradiation of the eye detector, using a bidimensional scan and a SOBP realized with the first six energies reported in Table 6. The target has been voxelized in 200×200×200 cells of $0.2\times 0.2\times 0.2\;{\text{mm}}^{3}$ volume. Irradiation was done simulating $5\times 10^{6}$ events.

For a more quantitative description of the deposited dose in the eye detector and the evaluation of the ratio between the dose deposited in the tumor and in the other eye components, a comparative dose‐volume histogram (DVH)^30^, ^31^ was built using the optimized setup described in Results section D. In Fig. 12, the integral DVH for the eye detector produced simulating $5\times 10^{6}$ events is shown. While 100% of the tumor volume resulted to be hit by an energy of about 45 MeV, the same energy was deposited only on 40% of the vitreous humor volume and all the other eye components had an even lower energy deposition in their volume (from 40% to 20% or less). In particular the optic nerve, the cornea, and the lens, the most dose sensitive subcomponents, received 45 MeV for 10% of their volume or less.

**Figure 13:**
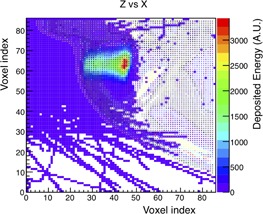
ZX projection of the irradiated eye in the human head reconstructed from DICOM images. Irradiation was performed with a point‐like beam of 100.51 MeV nominal energy and with a range shifter of 46 mm thickness positioned at 65 mm from the isocenter, simulating 10^5^ events. Voxel indexes, as read from DICOM files, go from 0 to 86 in both x‐ and z‐axes. The voxel has a volume of $0.97\times 0.97\times 2.0\;{\text{mm}}^{3}$. Voxel index number 43 corresponds to the isocenter position.

The DICOM detector was irradiated with a point‐like beam, with nominal energy of 100.51 MeV, degraded with a 46 mm thick PMMA range shifter, to let the beam stop inside the patient's eye where the tumor is located. In Fig. 13, the ZX projection of irradiated DICOM eye is shown. In the figure, the same voxelization for detector and irradiated cells is used.

## IV. DISCUSSION

The standard transport beamline of the CNAO facility has been entirely simulated, avoiding, when possible, any approximation. Different kinds of detectors were used within the simulation process, starting from simple water box or air layer detectors, up to very complex geometries, like an eye‐modeled detector (the “eye detector”) or an eye CT scan‐modeled detector (the “DICOM detector”).

The simulation was validated against experimental data by measuring the beam profile FWHM on the transversal plane and the proton depth dose curve in water. Simulation and experimental data were found to be in agreement, within the uncertainties, for both kinds of measurements.

Using an active lateral scanning irradiation with 3 mm steps and a SOBP achieved with an opportune number of beam energies, three‐dimensional irradiation uniformity within about 5% could be reached. To cut undesired energy depositions on healthy tissues, a 1 cm thick elliptical brass collimator was shaped around a simulated tumor inside of the eye detector. The use of the collimator improves the energy deposition within the irradiation central area and strongly reduces the lateral penumbra.

Adjusting the range shifter thickness to an optimized value of 43 mm and inserting a brass collimator of an appropriate aperture $\left(22\times 20\;{\text{mm}}^{2}\right)$, the dose deposited on the tumor could be highly enhanced with respect to the dose delivered to the surrounding normal structures, as shown by graphical representation of dose deposition and comparative DVH built for all the eye components in the eye detector. The statistics used to reproduce the DVH did not correspond to the true dose for each patient irradiation; however, the energy deposition behavior and the mutual dose sharing have been described in detail for each eye subcomponent. As a matter of fact, the typical dose for an eye treatment from the literature^(1)^ is of about 50‐60 Gy (RBE) fractionated over four to five days, where the RBE is set to 1.1. For the CNAO synchrotron, a number of protons per spill up to approximately $1\times 10^{10}$ can be delivered. Spill duration is 1 s, while the pause between two consecutive spills about 5 s. It corresponds to a dose rate typically around 1 Gy/spill or 10 Gy/min. This means that each ocular treatment session would take about 60 s — that is a reasonable irradiation time during which the patient could keep the correct eye position by gazing at a predefined fixed light.

## V. CONCLUSIONS

In this work, a general‐purpose transport beamline for actively scanned protons, adapted for the treatment of uveal melanoma, has been investigated using Monte Carlo simulations. The CNAO existing transport beamline should be slightly modified, using an optimized setup with additional passive elements. In particular, a PMMA range shifter has been introduced in the transport beamline to degrade the beam nominal energy to the correct value. Since the beam transversal dose profile is broadening as a consequence of both insertion of the range shifter and the existing air gap, the patient positioning is suggested to be at a minimal distance from the last passive element along the beamline (5 cm was the optimal value found). Moreover, a brass collimator with an individualized aperture should be used to reduce the lateral dose penumbra.

This simulation study shows that the general‐purpose proton beamline existing at CNAO, although optimized to try to meet the requirements for ocular treatments, would be able to deliver small fields at shallow depths with a lateral penumbra much wider than the one (1–2 mm as 80%–20% penumbra) achieved by most beamlines specifically dedicated to eye treatments.^(4)^ This seems to represent a severe limitation for very small tumors (a few millimeters) or lesions very close to critical structures, while its impact on the clinical outcome for less demanding scenarios needs to be further investigated.

The use of DICOM images to build the “DICOM detector” represents a starting point to better address the problem of irradiation of a human eye with the tools commonly available in medical imaging and it will be developed in the near future.

The Monte Carlo approach adopted in this study will be also useful in future as a core development to check the reliability of treatment planning systems for real clinical cases.

## ACKNOWLEDGMENTS

We would like to thank the Geant4 staff, in particular Dr. P. Cirrone, for providing the hadrontherapy example. We would like to thank also Dr. M. Donetti (CNAO Foundation) for the support and the specifications of the dose delivery system he gave us. We deeply thank Dr. L. Raffaele from CATANA (INFN‐LNS) for fruitful discussions. This work was financially supported by the INFN–Pavia in the framework of the Monte Carlo‐INFN Italian project.
